# Evolution in Medicine—The Use of Association

**Published:** 1895-01

**Authors:** W. H. Leonard

**Affiliations:** Minneapolis, Minn.


### EVOLUTION IN MEDICINE.—THE USE OF ASSOCIATION.

W. H. Leonard, M. D., Minneapolis, Minn.

It is worth the while to read the "Declaration of Principles" of the different associations for the protection, propogation, and evolution of the law of similars. Each is a different statement from the other in regard to the same principle. The first two, the A. I. H. and the I. H. A., have their history and uses, and there is no questioning the benefit to the profession at large.

Now comes a new association—the "Society of Homœopathicians," and may be called the Society of Homœopathicians

(Limited). This society, no doubt, will be useful in three directions—protection, propagation, and evolution, and it is to be hoped that the latter will not be the least, for some of the best thinkers speaking the English language are concerned in its success. The question arises, Why so many associations? Evolution is the method of progress. A fuller statement of truth may come out of it.

Is it possible to form an enduring organization from a single principle? All principles must be dual, tri-une, or four-fold, and must be in mutual relation with cognate ones. This is the case with all living things, and principles must be living, or they are not principles.

Now, is this third "Declaration of Principles" any improvement on the former? It may be questioned. Let us arrive at a four-fold statement of the power or force of the law, viz.: First, mechanical; second, chemical; third, electrical, and fourth, spiritual, or, better for our purpose, psychical.

In sections I and III is mentioned a spirit-like life force. This must be referred to our fourth element. Nowhere in the six sections is there a hint of any force but the psychical or spiritual, except in the sixth. It is a question whether the Newtonian law of motion is sufficiently ample to express the basis for the formation of a medical society, even though limited to fifty. Can the law of cure be founded upon a mechanical law of motion? Does Newton's law of motion express the action of all force?

The cause of the formation of this society is doubtless laudable, but it is upon ethical grounds. The ethical has not been known in medicine, or certainly not made practical. There is wanted a statement which shall cover this point. The four-fold relations of the elements of an organic cell may be expressed: 1st, Physical; 2d, Intellectual; 3d, Ethical; and 4th, Spiritual.

Let there be a formation of another society of fifty or more, based upon a full scientific statement of principles underlying the profession of medicine, then will progress be through evolution.

